# Curcumin-Loaded Bacterial Cellulose/Alginate/Gelatin as A Multifunctional Biopolymer Composite Film

**DOI:** 10.3390/molecules25173800

**Published:** 2020-08-21

**Authors:** Nadda Chiaoprakobkij, Thapanar Suwanmajo, Neeracha Sanchavanakit, Muenduen Phisalaphong

**Affiliations:** 1 Biomedical Engineering Program, Faculty of Engineering, Chulalongkorn University, Bangkok 10330, Thailand; naddakij@hotmail.com; 2 Department of Chemical Engineering, Faculty of Engineering, Chulalongkorn University, Bangkok 10330, Thailand; 3 Centre of Excellence in Materials Science and Technology, Department of Chemistry, Faculty of Science, Chiang Mai University, Chiang Mai 50200, Thailand; thapanar.s@cmu.ac.th; 4 Center of Excellence for Regenerative Dentistry, Department of Anatomy, Faculty of Dentistry, Chulalongkorn University, Bangkok 10330, Thailand

**Keywords:** bacterial cellulose, alginate, gelatin, curcumin, biomaterials

## Abstract

Multifunctional biopolymer composites comprising mechanically-disintegrated bacterial cellulose, alginate, gelatin and curcumin plasticized with glycerol were successfully fabricated through a simple, facile, cost-effective mechanical blending and casting method. SEM images indicate a well-distributed structure of the composites. The water contact angles existed in the range of 50–70°. Measured water vapor permeability values were 300–800 g/m^2^/24 h, which were comparable with those of commercial dressing products. No release of curcumin from the films was observed during the immersion in PBS and artificial saliva, and the fluid uptakes were in the range of 100–700%. Films were stretchable and provided appropriate stiffness and enduring deformation. Hydrated films adhered firmly onto the skin. In vitro mucoadhesion time was found in the range of 0.5–6 h with porcine mucosa as model membrane under artificial saliva medium. The curcumin-loaded films had substantial antibacterial activity against *E. coli* and *S. aureus.* The films showed non-cytotoxicity to human keratinocytes and human gingival fibroblasts but exhibited potent anticancer activity in oral cancer cells. Therefore, these curcumin-loaded films showed their potential for use as leave-on skin applications. These versatile films can be further developed to achieve desirable characteristics for local topical patches for wound care, periodontitis and oral cancer treatment.

## 1. Introduction

Over the past years, various types of biopolymers have been studied and proposed as alternatives for biomedical approaches. However, there are still attempts to develop functional biomaterials to meet the increasing demands for practical and safe options for clinical uses. Antimicrobial biomaterial obtained by incorporation of antimicrobial agents into biocomposites shows promise as a useful and environmentally friendly method; however, antimicrobial agents such as antiseptics might cause some drawbacks as they can lead to bacterial resistance with prolonged use, allergic reactions and side effects with high dose usage. Recently, silver nanoparticles (AgNPs) have been one of the prevalent metallic materials being studied, owing to their antimicrobial properties. Despite potential as antimicrobial agents, AgNPs have been reported to possibly be harmful to human health. The cytotoxic characteristics of AgNPs against mammalian cells have been documented [1,2,3]. For example, silver and gold nanoparticles were found to reduce fibroblasts migration in vitro [4]. Long-term studies are required to assess the safety for humans of metal nanoparticle-based biomaterials. On the other hand, natural-based compounds have been popular in modern therapy due to the low cost, limited adverse effects and high efficacy. Bacterial cellulose (BC) is an extracellular polysaccharide excreted by bacteria. Despite the identical chemical formula to that of cellulose from plants, BC is free of lignin and hemicellulose. Several bacterial cellulose-based biocomposites have been studied as alternative biomedical materials. Disintegrated BC (in gel suspension form) is an alternative form for producing BC composite films for large-scale production. Disintegrated BC can behave as a reinforcing agent or binder to the composites; the resulting composite showed better performance and improved mechanical strength and stiffness [5]. Alginate (A) is a natural polysaccharide that is biocompatible, biodegradable and non-toxic. Alginate is not only biocompatible and gellable under mild conditions but also able to improve compatibility, homogeneity, binding and dispersibility of polymer blends [6]. However, it does not present binding sites for cell attachment to which mammalian cells can bind. Gelatin (G) is one of the promising biomaterials because of its biological origin from collagen with excellent biocompatibility. Gelatin contains Arg-Gly-Asp sequences that are crucial for cell proliferation. Curcumin (C) is a yellow, non-water-soluble bioactive agent extracted from *Curcuma longa*. In recent years, it has been well documented in many in vitro and in vivo studies that curcumin possess a wide range of beneficial properties including wound healing, along with anti-inflammatory, antioxidant, antimicrobial and anticancer properties [7,8,9]. Recently, there have been reports on loading curcumin into polymers to produce functional polymer composites for various approaches. Cellulose/curcumin films were developed by using 1-allyl-3-methylimidazolium chloride (AMIMCl) for the dissolution of cellulose and curcumin and fabricated via by casting method [10]. Nanocurcumin prepared by ultrasonic process were impregnated into gelatin cellulose fibers to form microbial resistant fibers [11]. Chitosan/cellulose microcrystal films incorporated with curcumin were fabricated by vapor induced phase inversion (VIPI) [12]. Antibacterial curcumin/PVA/cellulose nanocrystal films were fabricated as antimicrobial wound dressing film [13]. Curcumin were entrapped in nanocellulose/chitosan hydrogel by swelling equilibrium method [14]. Curcumin was incorporated into nanocellulose by using Tween 20 as a surfactant for delivery of curcumin to stomach and upper intestinal tract [15]. Curcumin loaded bacterial cellulose films was developed and reported for anticancer against malignant melanoma skin cancer cells [16]. Despite a number of benefits, instability under physiological condition could limit the efficacy of curcumin [17].

In our previous study, homogenized BC/alginate/gelatin (BCAGG) composite films were successfully fabricated [18]. This current work is intended to broaden the implementation feasibility of multifunctional BCAGG composites by directly loading curcumin into the polymer matrix during the film fabrication via a casting method. Film mechanical properties, fluid uptake ability in various media, surface wettability, bioadhesive residence time and antibacterial activity were thoroughly evaluated. In addition, evaluation as an oral wound dressing, cytocompatibility and proliferation of human keratinocytes (HaCaT), human gingival fibroblasts (GF) and oral cancer cells (CAL-27) on the film surface were preliminary assessed.

## 2. Results

### 2.1. Morphology

**Figure 1:**
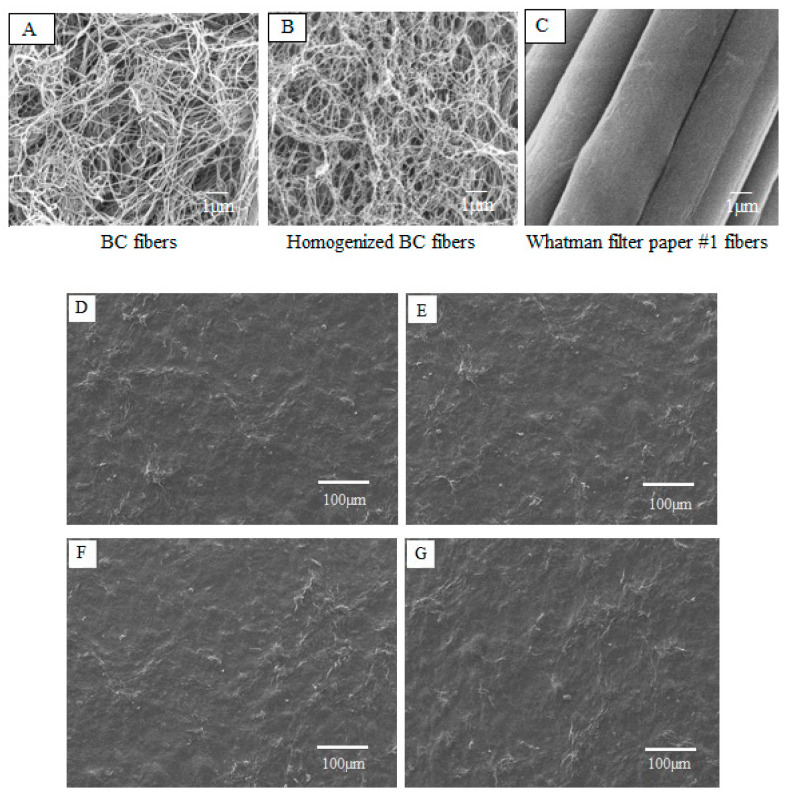
SEM images of: (**A**) BC fibers; (**B**) mechanically-disintegrated BC fibers; and (**C**) Whatman filter paper No. 1 fibers at 10,000× magnification. Surface morphology of: (**D**) BCAGG-C1; (**E**) BCAGG-C2; (**F**) BCAGG-C3; and (**G**) BCAGG-C4.

The morphology of BC, homogenized BC and plant cellulose (Whatman filter paper No. 1) fibers were depicted by SEM, as presented in Figure 1A–C. The micrometer scale SEM images show no significant difference between mechanically-disintegrated BC fibers and native BC fibers. The fibrillated BC appeared highly entangled and formed a web-like network structure (Figure 1B) with fiber diameter in nanometers and length in micrometers. Figure 1D–G shows the surface morphology of curcumin-loaded films of BCAGG-C1, BCAGG-C2, BCAGG-C3 and BCAGG-C4 (containing curcumin at 0.17 ± 0.3, 0.34 ± 0.2, 0.55 ± 0.2 and 0.75 ± 0.1 mg/cm^3^, respectively). It was observed that the entire surface was rather rough, with uniform appearance and no cracking. No significant microstructural differences existed for any film samples in this study.

### 2.2. FT-IR Analysis

**Figure 2:**
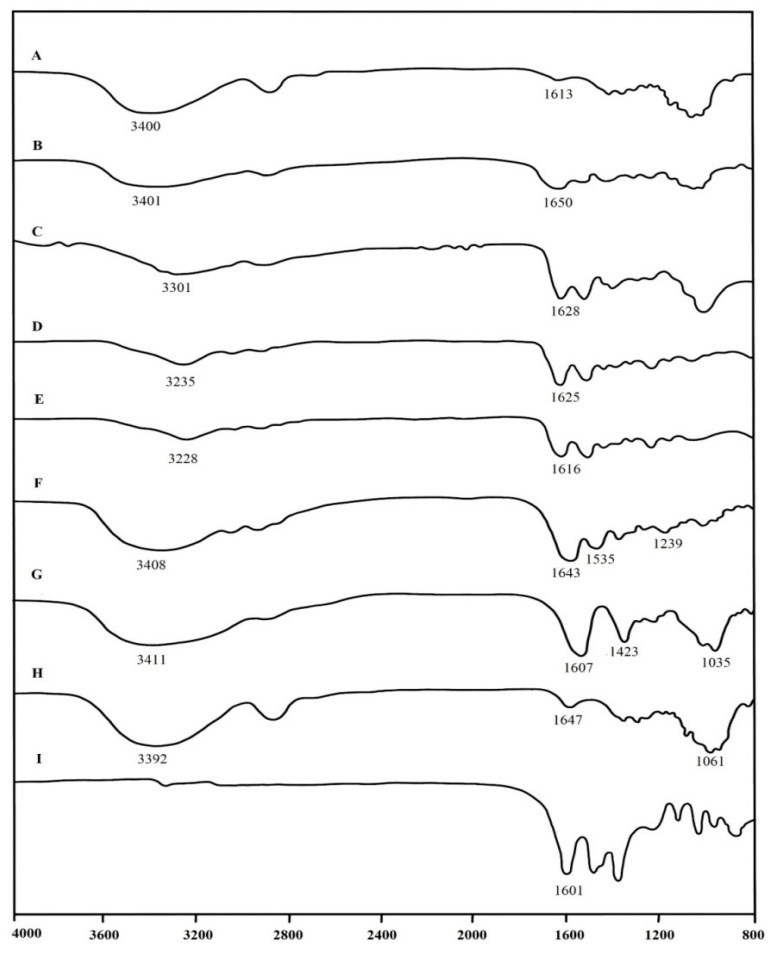
FT-IR spectra of: (**A**) BCAGG; (**B**) BCAGG-C1; (**C**) BCAGG-C2; (**D**) BCAGG-C3; (**E**) BCAGG-C4; (**F**) gelatin; (**G**) alginate; (**H**) BC; and (**I**) curcumin.

The interactions among the components of samples were investigated by FT-IR spectra (Figure 2). The typical absorption bands of BC were –OH groups at 3392 cm^−1^, H-O-H bending of absorbed water at 1647 cm^−1^ and C-O-C stretching at 1061 cm^−1^ [18]. For FT-IR spectrum of alginate, the peaks at 3411, 1607 and 1423 cm^−1^ were attributed to –OH stretching, -COO- asymmetric stretching and -COO- symmetric stretching, respectively. For gelatin, the peak at 3408 cm^−1^ was attributed to the partially overlapped stretching vibrations of O-H and N-H groups. The amide I (C=O (stretching, amide II (N-H) bending and amide III (C-H) stretching, characteristic peaks of gelatin (protein), were clearly observed at 1643, 1535 and 1239 cm^−1^, respectively. The major peaks of interest for curcumin were at around 3400 and 1601 cm^−1^. FT-IR spectra of pure curcumin showed a peak at 3413 cm^−1^, denoted as phenolic O-H stretching, and sharp peaks at 1601 cm^–1^, indicative of C=C benzene stretching vibrations.

The FTIR results of all of curcumin-loaded films displayed all the characteristic bands of BC, alginate, gelatin and curcumin components. The result indicates their bonding interactions. Broad absorption bands between 3200 and 3550 cm^−1^ were due to overlapping of –OH groups and -NH_2_ groups stretching vibrations [19]. The peaks of –OH stretching in the spectrum of curcumin-loading composites were obviously shifted and intensity changed in the range of 3200–3500 cm^−1^ as compared to that of BCAGG. This is an indication of intermolecular interaction between phenolic groups of curcumin and BCAGG matrix; therefore, curcumin likely interacted via hydrogen bonding interaction in the polymer matrix. The peaks at 1650, 1628, 1625 and 1616 cm^−1^ of all curcumin-loaded composites were assigned to the C=C stretching vibration of benzene rings, regarded as a characteristic peak of curcumin [20]. The major peaks of interest for curcumin are at around 3500 and 1601 cm^−1^. FT-IR spectra of curcumin showed a peak at 3513 cm^−1^, denoted as phenolic O-H stretching, and sharp peaks at 1601 cm^−1^, indicative of C=C benzene stretching vibrations [21].

### 2.3. Fluid Absorption Capacity

**Figure 3:**
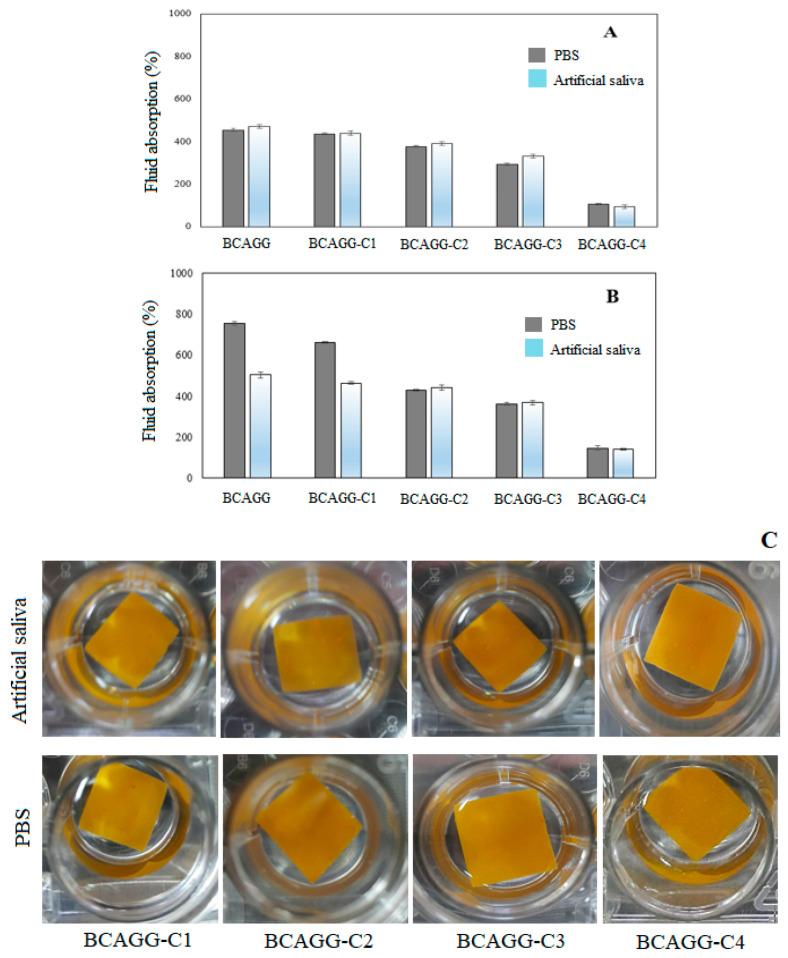
Fluid absorption after the immersion at 37 °C in PBS (pH 7.4) and artificial saliva (pH 6.2) for (**A**) 6 h and (**B**) 24 h; and (**C**) film samples after the immersion for 48 h.

The samples were completely submerged into PBS and artificial saliva in an effort to simulate clinical conditions. Fluid uptake capacity is an important factor in maintaining the optimum moist environment at the target site. Figure 3A,B shows that the fluid uptake ability decreased with an increase in curcumin contents in both PBS and artificial saliva. During the first 6 h, the fluid uptake ability in artificial saliva solution was slightly higher than that of PBS buffer uptake ability. The fluid uptakes increased rapidly and reached maximum after 24 h of incubation. After 24 h of incubation, BCAGG and BCAGG-C1 showed relatively higher PBS absorption compared to artificial saliva (Figure 3B). The fluid absorptions were in the range of 100–700%. All samples were able to maintain integrity throughout the 48 h of immersion (Figure 3C). The release of curcumin was not observed in the testing mediums during the experimental study. The amount of released curcumin was determined by UV-Vis spectrophotometry (Shimadzu UV-2550, Tokyo, Japan) at the wavelength of 420 nm. All tests were done in triplicate. The results show that there was no curcumin in the testing mediums. This is similar to observations of the previous report of curcumin loaded in gelatin/curcumin films, in which curcumin did not diffuse out of the polymer matrix [22]. Due to the high entanglement of mechanically-disintegrated BC fibers and good colloidal properties of BC/alginate/gelatin blend [23], curcumin was well embedded into the matrix. In this study, curcumin was entrapped inside the cross-linked BCAGG network. During the drying process, water evaporated, and a dense and stable structure of films was formed and entrapped curcumin within the matrix. The FTIR result also indicate an intermolecular interaction between phenolic groups of curcumin and BCAGG matrix. Because curcumin has poor solubility in water, it hardly dissolves in water-based mediums. Therefore, curcumin was not released from the BCAGG-C films into PBS and artificial saliva.

### 2.4. Surface Wettability

**Figure 4:**
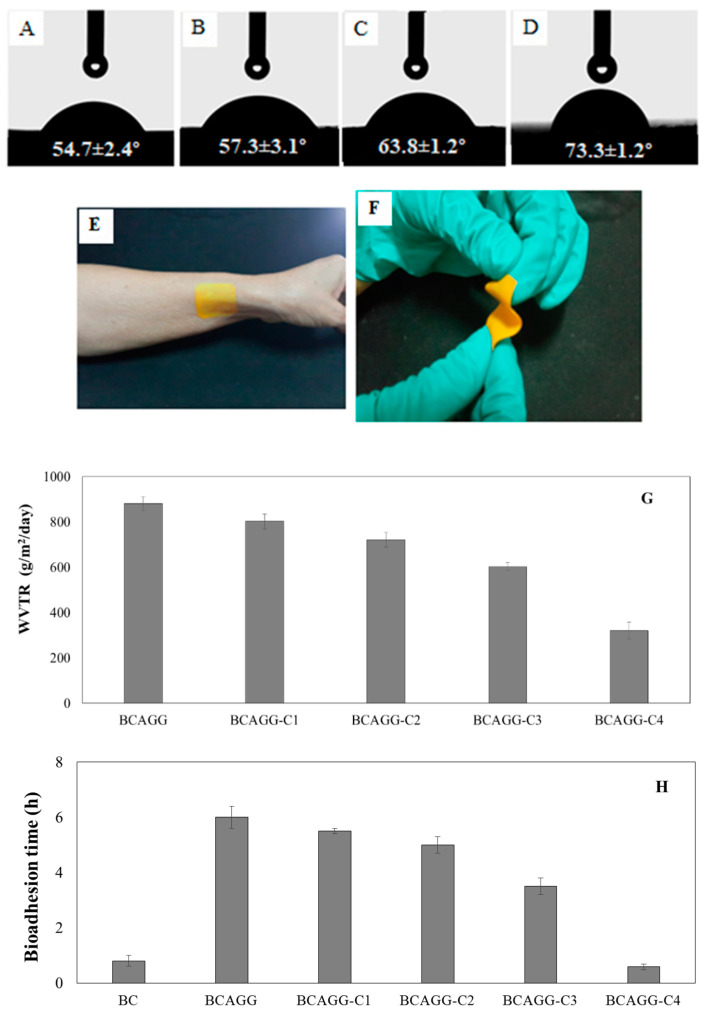
Water contact angles of: (**A)** BCAGG-C1; (**B**) BCAGG-C2; (**C**) BCAGG-C3; and (**D**) BCAGG-C4; (**E**) film attachment to skin; (**F**) film flexibility; (**G**) WVTR; and (**H**) bioadhesion time.

The water contact angle of BCAGG was 49.5° [23], whereas those of BCAGG-Cs were 54.7–73.3°. The water contact angle of curcumin-loaded films increased with increasing curcumin content, as shown in Figure 4A–D. It was found that all hydrated films provided perfect coverage and neatly adhered to the skin with good flexibility, as shown in Figure 4E,F, respectively. 

### 2.5. Water Vapor Transmission Rate (WVTR)

Figure 4G shows that WVTR of curcumin- loaded films decreased with the addition of curcumin. WVTRs of BCAGG-C films were around 300–800 g/m^2^/24 h.

### 2.6. Bioadhesion Time

Bioadhesion times of BCAGG and BCAGG-C films are shown in Figure 4H. BCAGG exhibited more hydrophilic surface, leading to a faster rate of water absorption and higher adhesive performance compared with the curcumin-loaded films. It was observed that the adhesion time decreased with the addition of curcumin. The adhesion of the composite films lasted 0.5–6 h.

### 2.7. Mechanical Properties

**Figure 5:**
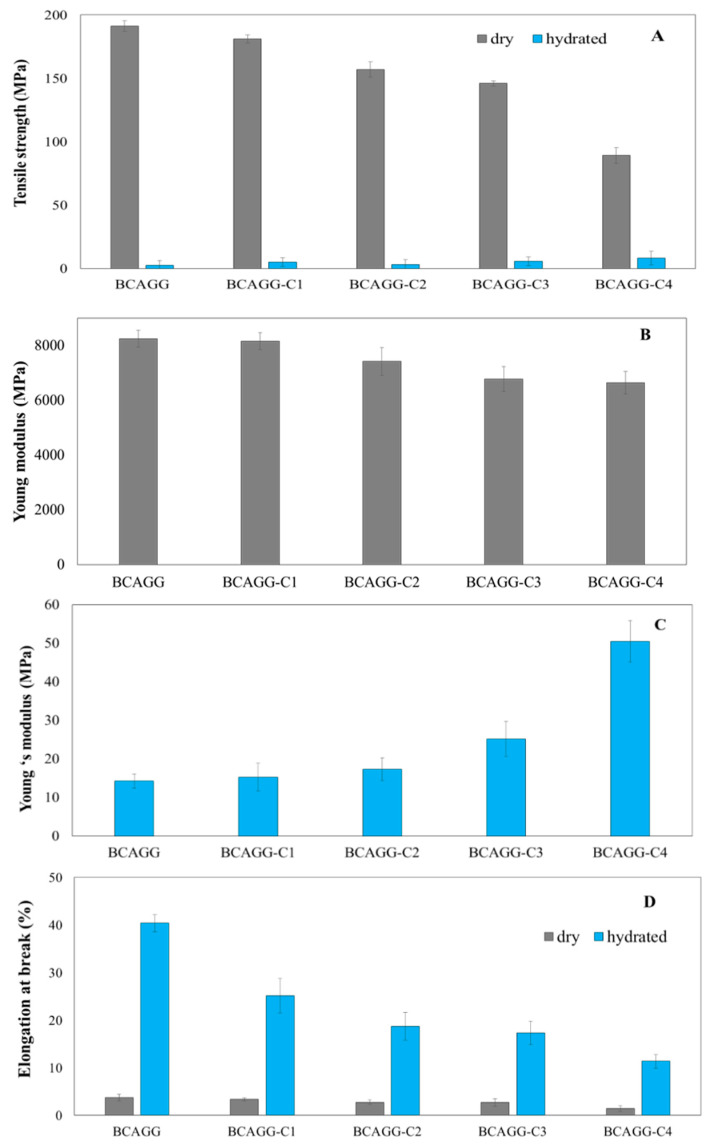
Mechanical properties of films: (**A**) tensile strength of dry and hydrated films; (**B**) Young’s modulus of dry films; (**C**) Young’s modulus of hydrated films; and (**D**) elongation at break (%) of dry and hydrated films.

The mechanical properties of dry and hydrated samples are shown in Figure 5. Tensile strength and Young’s modulus of the dry films were in the range of 80–170 and 7000–8000 MPa, respectively, and the mechanical properties of the films decreased with increase in curcumin content. The mechanical strengths were much lower in the wet state; however, it was observed that tensile strength and Young’s modulus of hydrated curcumin-loaded films relatively increased in comparison to non-curcumin loaded ones. Elongation at break of the films in both dry and wet states (Figure 5D) decreased with curcumin loading content. Elongation at break of the hydrated films was in the range of 15–40%.

### 2.8. In Vitro Cell Studies

**Figure 6:**
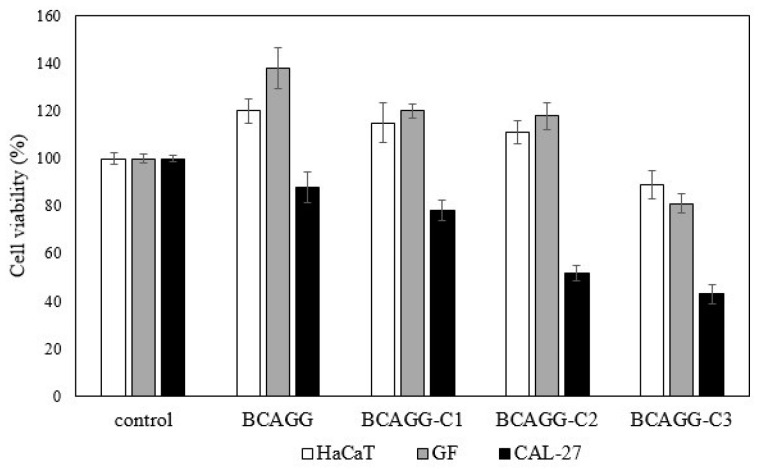
Cell viability (%) of HaCaT, GF and CAL-27 at 48 h after seeding.

**Figure 7:**
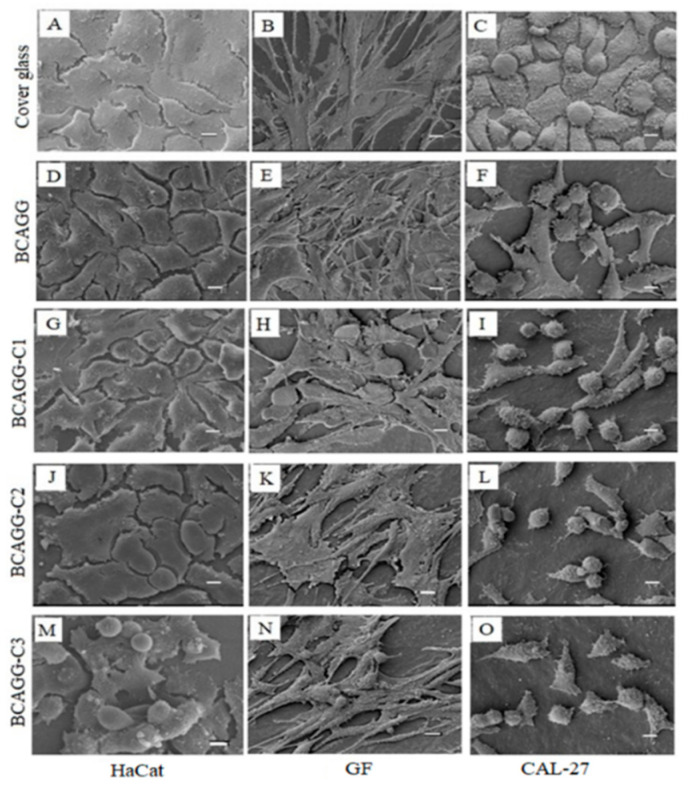
SEM images of HaCaT, GF and CAL-27 on film samples for 48 h (scale bar = 10 µm)**.**

The direct culture method was adopted to evaluate in vitro cytotoxicity of the fabricated films. Cell viability after cultivation for 48 h is shown in Figure 6. All samples were nontoxic to normal healthy cells (HaCaT and GF). According to ISO10993-5, the materials are classified as a noncytotoxic material if cell viability is greater than 70%. An increase in cell number on BCAGG, BCAGG-C1 and BCAGG-C2 was observed at 48 h incubation time. Along with curcumin loading, CAL-27 cell viability on BCAGG-C2 and BCAGG-C3 decreased by almost half in comparison to the control, which appears to be due to the anticancer property of curcumin. Morphologies of HaCaT, GF and CAL-27 are shown in Figure 7. HaCaT and GF cells tended to grow in flat monolayer with cells oriented in a parallel manner on coverslips. HaCaT and GF cells spread well on the fabricated films and retained their structural and morphological characteristic of cells. HaCaT exhibited and maintained the polygonal cell shape, which is typical of HaCaT cell morphology. GF cells tended to be randomly oriented, elongated and growing into multiple layers. Increasing curcumin content, to some extent, induced some cellular morphology changes. As shown in Figure 7, some GF cells exhibited a short spindle-shaped morphology on BCAGG-C3 film. Some round cells of HaCaT were also observed on BCAGG-C3. Meanwhile, CAL-27 cells displayed distinct morphological changes, including cell shrinkage, rounding up, irregularity in shapes, ruffled cell membrane surface and partial detachment on BCAGG-C films. CAL-27 cells were isolated, with the presence of cellular debris.

### 2.9. Antibacterial Tests

**Figure 8:**
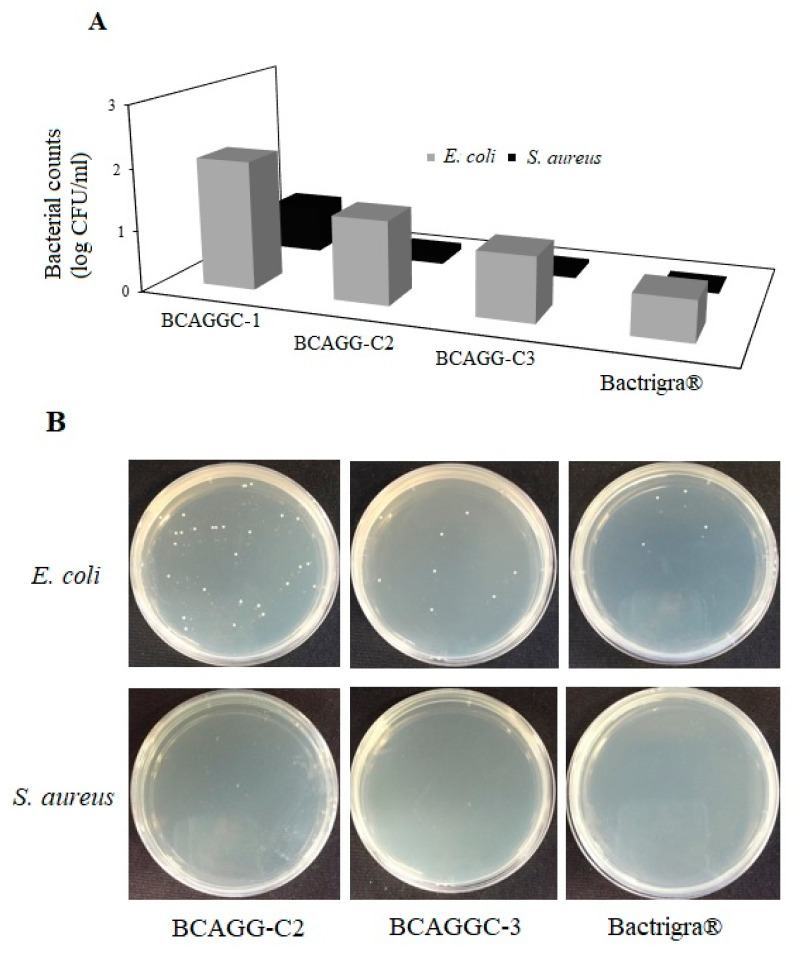
(**A**) Number of viable bacteria cells on the sample surface; and (**B**), agar plates of *E. coli* and *S. aureus* colonies at 48 h incubation on BCAGG-C2, BCAGG-C3 and Bactrigra^®^.

The results of antibacterial tests are shown in Figure 8A,B. BCAGG-C1, BCAGG-C2 and BCAGG-C3 films as well as commercially available antibacterial wound dressing, Bactrigra^®^ were tested against *E. coli* and *S. aureus.* From our preliminary study, BCAGG had no antibacterial activities. By loading curcumin into the biocomposite matrix, the antibacterial activities against *E. coli* and *S. aureus* were enhanced with the increase in curcumin concentration. The bacterial counts of *E. coli* and *S. aureus* cells from 48 h incubation on surface of BCAGG-C3 were comparable to those of the commercial wound dressing. It was noticed that the composite films had greater inhibition effect against Gram-positive bacteria (*S. aureus*) than Gram-negative bacteria (*E. coli*).

## 3. Discussion

The fibrillated BC appeared highly entangled and formed a web-like network structure. The high aspect ratio fibers were suitable for applying as a reinforcing agent in composite materials [23]. BCAGG films demonstrated a uniform structure. According to SEM analysis, there was no significant effect of curcumin loading on the surface morphology. A good distribution of curcumin without agglomerations was obtained. This suggests reasonably good compatibility between curcumin and other polymers in the matrix.

The FTIR results of all curcumin-loaded films show characteristic absorption bands of curcumin, suggesting that the films should retain the functions of curcumin. It was found that there were no additional peaks, excluding the specific bands of BC, alginate, gelatin and curcumin. However, typical peaks of curcumin-incorporated film were shifted to some extent as compared to BCAGG film. The results are in accordance with the previous report [24]. Similar observations were reported in gelatin/curcumin composite films [25], in which the FT-IR and NMR results indicate the hydrogen bonding between curcumin and gelatin. According to the previous work of BC–alginate matrix, the slight shift and intensity changes were observed due to intermolecular hydrogen bonding formation between anionic chains of BC and alginate [6]. The opposite charge of polyanionic polysaccharide chains and positively charged gelatin could electrostatically attract each other, leading to the formation of stable polyelectrolyte complexes [18]. These findings were consistent with the previous study on complex formation between oppositely charged macromolecules [26]. The amino groups of gelatin and carboxyl groups of alginate had electrostatic interactions with each other. In addition, it was reported that cellulose could form hydrogen bonding with alginate and gelatin within the network and enriched a dense and rigid network structure [27]. Curcumin-loaded BCAGG depicted similar characteristic peaks of all parental molecules. The results show that there was no new peak generated or disappeared after the addition of curcumin. However, small shifts and intensity changes were observed, which might be attributed to local modifications leading to small variations of rotation and vibration frequencies via hydrogen bonds. During the drying step, a dense and stable structure of films was formed and curcumin was entrapped inside the cross-linked BCAGG network. In addition, it was found that the peaks of –OH stretching in the spectrum of curcumin-loading composites were obviously shifted and intensity changed in the ranges of 3200–3500 and 1650–1616 cm^−1^ as compared to that of BCAGG, which indicated the intermolecular interaction between phenolic groups of curcumin and BCAGG matrix. Therefore, curcumin likely interacted via hydrogen bonding in the polymer matrix. The negatively charged groups could interact with –OH groups of curcumin through hydrogen bonding [28].

The fluid uptake ability of the films, which decreased with the increase in curcumin loading, can be attributed to hydrophobic property of curcumin. It was also found that curcumin did not diffuse out of the films throughout 48 h immersion in PBS and artificial saliva. It was previously reported that cellulose crystals acted as a diffusion barrier that retarded the release of curcumin from the film samples [12]. Hydrogen bonding interactions within the film matrix restrict the relaxation and motion of polymeric chains. Such physical crosslinks would impede water molecules diffusion and retard curcumin release. At the beginning, the fluid uptake ability in artificial saliva solution was slightly higher than that of PBS buffer. The fluid of artificial saliva, which contains carboxymethyl cellulose (CMC) as a viscosity modifier to mimic human saliva, had better wetting properties on the material surface. CMC is commonly known as humectant, which is a substance that actually bonds with water molecules to increase the water content to itself. The fluid uptakes increased rapidly and reached the maximum after 24 h of incubation. Alginate does not swell in acidic environments but possesses hydrophilic and water retaining properties in neutral or mildly alkaline environments [29]. Gelatin/polyglycerol sebacate copolymer hydrogels were also found to swell less under acidic pH conditions [30]. Similar behavior was also reported for calcium alginate/chitosan bi-polymeric beads in acidic stimulating intestinal fluid [31]. Under acidic conditions, the amino groups were protonated and the electrostatic interactions between carboxyl groups (COO-) within the polymeric network and protonated amine groups (-NH_3_+) were strengthened, resulting in a denser structure with lower water uptake. Consequently, the films without or with low curcumin loading (BCAGG and BCAGG-C1) exhibited higher PBS absorption than that of artificial saliva after the 24 h immersion in the fluids.

Typically, wound pH values vary from 5.5 to 7.8. After prolonged periods, it will settle at pH 7.4–7.0 [32]. Wound dressing absorption capacity is important for wound healing. According to the study of absorption capacity of commercial super-absorbent dressings [33], the fluid absorption capacity was found in a range of 50–220%. The curcumin-containing films developed in this study were classified as thin film. It was demonstrated that the films could adhere and stick directly to skin (without adhesive) with high fluid uptake ability in the range of 100–700%.

The composite films became more hydrophobic with the addition of curcumin. Generally, surfaces with a water contact angle of less than 90° are considered hydrophilic, while those with a water contact angle greater than 90° are considered hydrophobic. To create sufficient film adhesion, liquid has to both wet the contact surface and flow easily over the entire surface. Topical drug carrier films should be capable of adhering to surfaces in order to bring drugs in contact with the target site for a sufficient period of time. Surface wettability also influences protein adherence, as well as cell attachment and cell proliferation, and it is one of the critical factors in determining cell behavior. Based on the hydrophilic properties, all hydrated films in this study provided perfect coverage and were able to neatly adhere to the skin. The composite films were also able to cope with curves and uneven textures.

Diffusion of water vapor through films affects the barrier properties of those films. Topical films in biomedical applications should be occlusive to bacteria and viruses; however, water vapor, oxygen and carbon dioxide should be easily exchanged. Films are supposed to allow moisture to evaporate and to maintain normal skin function under the films. High WVTR films lead to quick moisture loss, and they cause wound dryness, while low WVTR might cause exudate storing, which may retard the wound healing process [34]. Intact human skin WVTR ranges 240–1920 g/m^2^/24 h, whereas the WVTR of an uncovered wound is around 4800 g/m^2^/24 h [35]. WVTR values of existing commercial film and hydrocolloid dressings range between 436 and 900 g/m^2^/24 h [36,37]. Injured skin (first degree to third degree burns) can range from 278.4 to 4274 g/m^2^/24 h [38]. The WVTR characteristic of the fabricated films in this study was found to be comparable to human skin and commercial wound dressings.

Adhesion of film to the surface is an important key factor for retaining the film on the target site. It was demonstrated that the composite films presented good adhesion properties and maintained their integrity on the target sites. For the further application as oral wound dressing, the films were submerged into artificial saliva. It was found that hydrated films adhered well to the mucosa surface, and the lack of air between surfaces was attributed to good surface wettability. A vacuum system is formed and the external air pressure keeps the material and surface together, allowing the films to maintain intimate contact. The functional groups of hydrophilic polymers such as hydroxyl, carboxyl and amine groups are able to form hydrogen binding between the material and superficial surfaces [39]. In addition, positive charges of gelatin molecules were able to form electrostatic bonds with the negative charge of mucosal surfaces. Therefore, the developed films can be applied as adhering films for the oral cavity. Once films are place in the mouth, they adhere strongly, maintaining moisture without causing drying. Previously, the in vitro adhesion residence time of alginate-based mucoadhesive bi-layered buccal patches for antimigraine treatment reportedly varied from 20 to 25 min [40]. Another study on mucoadhesive patches containing a miconazole drug reported that the adhesion residence time was 2 h [41]. It has been shown that the fabricated film in this work has advantages in terms of flexibility and long bioadhesion residence time.

The mechanical properties of the films decreased with the increase in curcumin content. This was possibly due to the disruption of hydrogen bonding between BC fibrils by the addition of an interfering compound and a decrease in intermolecular interactions between polymeric backbone chains and the presence of additives. The dispersed curcumin particles restrict the motions of polymeric chains, thus reducing their strength and elongation tendencies. Similar patterns of changes in mechanical properties have been observed in other studies. For example, mechanical properties were reduced by the integration of turmeric extract into chitosan films [19]; tensile strength, Young’s modulus and elongation at break all tended to decrease as a function of increasing curcumin loading in BC film [16]; and phenolic–protein inter-chain interaction resulted in a decline in flexibility of gelatin films [42].

Mechanical behavior after hydration is also important in predicting the in vivo clinical performance of the films. All samples in hydrated condition were found to be softer and more flexible compared to samples tested in the dry condition. Those results suggest that hydration of samples before use could provide the samples with more flexibility. Under hydrated condition, clusters of water molecules could fill intervening voids in the matrix. Water behaved as a plasticizer by creating bonds with hydrophilic groups of the polymers resulting in the increase of ductility. It was observed that mechanical strength of hydrated curcumin-loaded films increased in comparison to non-curcumin loaded ones. Curcumin solid content increased rigidity of the polymeric matrix. Curcumin molecules partially limit the diffusion of water molecule into the matrix. This is possibly due to interlocking effects, such as micro-pins within the polymeric matrix [43]. Curcumin restricts the segmental motions of the polymer chains, leading to the increase in matrix stiffness. Under wet environment, water molecules penetrated the composite and diffused along fiber–matrix interfaces causing debonding between fiber and matrix [44]. Mechanical properties showed that the fabricated films possessed the appreciable strength and were conformable enough for practical use. This good balance between stiffness and elasticity needs to be achieved for the clinical approach. Film should be stiff enough to bear forces and maintain its integrity during clinical uses. Meanwhile, a very stiff film does not allow for good clinical utilization. The design and adoption of leave-on skin applications should be a good match with natural skin in terms of mechanical strength and flexibility. The oral mucosa possessed a wide range of possible elastic modulus from 0.06 to 8.89 MPa [45]. Typically values of 1–5 MPa were adopted for simulations [46]. Tensile strength and Young’s modulus of commercial wound dressings are 0.9–28.3 and 0.08–8.85 MPa, respectively [47,48]. Based on the mechanical characteristics exhibited by the commercial benchmark, BCAGG, BCAGG-C1, BCAGG-C2 and BCAGG-C3 have appropriate mechanical characteristics for application as wound dressings.

Overall, cell growth of HaCaT and GF exhibited consistent trends without great differences among all samples. Both HaCat and GF cells tended to orient themselves and presented numerous cellular extensions and 3D-like structure that more closely resemble in vivo tissue. The collagen fibers in the wound tissue will organize along the stress lines of injury. The collagen fibers in scar tissues are arranged in parallel to the skin surface, as opposed to a non-parallel conformation in healthy skin [49]. Matrix stiffness plays an important role in the cell signaling that regulates cell behavior. Substrate rigidity regulated cancer cell invasiveness [50]. Breast cancer cells exhibited increased proliferation and migration through stiffer surface [51]. The migration speed of U-474 MG human glioblastoma cancer cells increased as substrate stiffness increased [52]. In most models of cell spread, cells must generate traction forces at adhesion sites so that cells can be stretched. Traction forces are not able to form without adequate external resistance from a soft substrate. In this study, CAL-27 cell viability gradually decreased on non-curcumin loaded BCAGG. Possibly, stiffness-related effects prevailed over hydrophilic and surface properties. The number of CAL-27 cells significantly dropped in curcumin-loaded BCAGG. This was attributed to the synergistic effect of stiffness and addition of curcumin. It was postulated that functional adaptation was acquired by the cancer cells to survive and thrive in the altered environment. Curcumin has anticancer activity against oral squamous cell carcinoma (OSCC) via both autophagy and apoptosis [53]. It has been suggested that, during cellular stress, to survive starvation or environmental toxicity, cells utilize autophagy to adapt to the microenvironment. Autophagy due to excessive stress could lead to cell death. According to our previous report, the release of curcumin from curcumin-loaded BC films showed strong cytotoxic effect against A375 human melanoma cancer cells, without significant cytotoxic effect against human keratinocytes and human dermal fibroblasts [16]. In this study, although no significant release of curcumin was observed in PBS and artificial saliva, curcumin-loaded BCAGG films (BCAGG-C2 and BCAGG-C3) showed cytotoxicity against oral cancer cells (CAL-27), but non-cytotoxicity to HaCaT and GF cells. Since curcumin was not released, the regulation of this response should be from the contact and interaction of curcumin to some kinds of cell surface molecules, which then modulated the cell behaviors. Recent studies demonstrated that curcumin can bind to cell membrane-bound toll-like receptors (TLRs) [54,55]. Curcumin demonstrates an antagonist effect on the TLR2 and TLR4 downstream signaling pathway resulting in an anti-inflammatory effect or induction of cell apoptosis [54]. TLR2 and TLR4 are found in several cell types including oral epithelial cell, gingival fibroblast and with overexpression by oral cancer cells [56,57,58]. Curcumin effectively modulates the TLR response and thereby exerts its potent therapeutic effects against a range of diseases such as inflammation, infection and autoimmune diseases including cancer [59]. Curcumin also has abilities to induce cell apoptosis, suppress the expression of multiple oncogenes and transcription factors inducing epithelial-to-mesenchymal transition (EMT) in cancer cells through interfering with the binding of Wnt to its cell surface receptor in the Wnt/β-catenin signaling pathway, suggesting curcumin as a potential target for anticancer therapies [60]. A further study should be performed find the actual mechanism on cancer viability of curcumin-loaded film.

The curcumin-loaded films had substantial antibacterial activity against *E. coli* and *S. aureus*. It was noticed that the composite films had greater inhibition effect against Gram-positive bacteria (*S. aureus*) than Gram-negative bacteria (*E. coli*) Several reports also indicate that curcumin is more effective on Gram-positive than Gram-negative bacteria. Bacteriostatic of gelatin/curcumin composite films showed stronger activity against Gram-positive than Gram-negative ones. Curcumin-loaded BCAGG films may possibly be identified as self-disinfecting, bacterial resistant composites as they did not accumulate bacteria growth over their surfaces. They could also be categorized as a passive antibacterial material as they did not release antimicrobial agents to the environment yet were able to inhibit bacterial growth. The alteration in antibacterial effect might be due to the difference in bacteria cell membrane structures. Gram-positive bacteria were found to be more susceptible to many natural antibacterial products [61,62]. Gram-positive bacteria have a thicker layer of peptidoglycan. On the other hand, Gram-negative bacteria have a cell wall composed of a thin layer of peptidoglycan surrounded by an outer membrane. The outer membrane of Gram-negative bacteria contains lipopolysaccharide, in addition to proteins and phospholipids [63]. The distinctive structure of the Gram-negative bacteria outer membrane could prevent some antimicrobial agents from entering to the cell. The results in this study demonstrate that curcumin exhibits some antibacterial activities despite being trapped in a biocomposite matrix of BCAGG. Auto-oxidation, one of the bactericidal properties of curcumin, could produce reactive oxygen species (ROS) such as hydrogen peroxide and superoxide, thereby disrupting integrity of the bacterial cell wall leading to cell death. Only the contact between the curcumin and bacterial cells is required for this mechanism as these free radicals have a short half-life [64]. Previously, it was shown that binding of curcumin, an amphipathic and lipophilic molecule, to the bacterial cell wall increases the cell membrane permeabilization of *S. aureus* and *E. coli* causing cell membrane damage and leading to cell lysis. This mode of antibacterial activity of curcumin on Gram-positive bacteria is more effective than that on Gram-negative bacteria according to the composition of cell wall [65]. However, further studies should be clarified for the definite mechanisms of action by the curcumin loaded film.

## 4. Materials and Methods

### 4.1. Preparation of Bacterial Cellulose/Alginate/Gelatin Films Incorporated with Curcumin

The preparation of BC/alginate/gelatin composite (BCAGG) was performed according to our previous work [23]. Films of BCAGG were fabricated from a blend of the BC, alginate and gelatin at a weight ratio of 60:20:20, added with glycerol at 2 g/10 g of gelatin solution. To prepare the curcumin-loaded BC/alginate/gelatin (BCAGG-C), solutions of curcumin (>95% purity, Sigma-Aldrich, St. Louis, MO, USA) at concentrations of 2, 4, 6 and 8 mg/mL were prepared by dispersing curcumin in absolute ethanol. Curcumin solutions of 1 mL were added into 100 g of BC/alginate/gelatin blends, and the mixtures were added with glycerol at 2 g/10 g of gelatin solution. All mixtures were thoroughly stirred together for 4 h until homogeneous mixtures were obtained. The mixtures were placed in a 14.5-cm diameter sterile Petri-dish (40 g/plate) and air-dried at room temperature for 24 h. After that, all dried films were cross-linked in a 1% (*w*/*v*) solution of calcium chloride for 1 h. The films were then rinsed with DI water and dried at room temperature (30 °C). The films were then placed in airtight containers at room temperature. BCAGG-C1, BCAGG-C2, BCAGG-C3 and BCAGG-C4 represented BCAGG-C prepared with 2, 4, 6 and 8 mg/mL curcumin solution, respectively. The actual curcumin content in each film was also determined. Each sample (2 cm × 2 cm) was immersed in 10 mL absolute ethanol and stirred at 100 rpm for 6 h and 1 mL of supernatant was then collected. The actual content of curcumin was quantified by UV-visible spectrophotometry (Shimadzu UV-2550, Tokyo, Japan) at the wavelength of 420 nm.

### 4.2. Characterization of Composite Films

Scanning Electron Microscope (SEM) was used to observe morphology of the samples. Films were cut and sputter-coated with gold in a Balzers-SCD-040 sputter coater (Balzers, Liechtenstein). Surface feature analysis was visualized using a JSM-5410LV scanning electron microscope (JOEL, Japan) operated at an acceleration voltage of 15 kV.

The information on chemical structural of the composite films was collected by FT-IR analysis using Spectrum One FT-IR spectrometer (PerkinElmer, Waltham, MA, USA). The dried samples were mixed with KBr and compressed into semitransparent KBR pellets before the measurement. The FT-IRs of samples were analyzed within the range of 4000–800 cm^−1^ at a scanning resolution of 2 cm^−1^.

Fluid uptake ability of the samples was measure by immersing the weighed samples in PBS (pH 7.4) and artificial saliva (pH 6.2) at 37 °C for 6 and 24 h. Excess fluid on the sample surface was removed by blotting out with tissue papers. The weights of wet samples were then recorded. All tests were done in triplicate. Fluid absorption capacity was calculated using the following equation:

$$\%\mathrm{WAC}=\;\frac{\left(\mathrm{Ws}\;-\;\mathrm{Wd}\right)}{\mathrm{Wd}}\;\times \;100$$

where Wd and Ws are the weights of dry sample and wet sample, respectively.

Surface wettability of the films was evaluated as static water contact angle using OCA 20 contact angle analyzer (Dataphysics, Filderstadt, Germany). Each of samples was placed on the platform of the contact angle analyzer just below the needle, from which a 2 μL water droplet was dispensed. The contact angle was measured in degrees using the SCA20 software. Measurements were performed at least 6 different locations for each sample.

WVTRs of samples were analyzed by water vapor permeability analyzer, PERMETRAN-W^®^ model 398 (Mocon, Minneapolis, MN, USA). The test condition followed ASTM E398-03 standard. The determination of WVTR was carried out at 38 °C and 98% relative humidity. The test specimen was sealed to the open mount of test dish containing a desiccant and the assembly placed in a controlled atmosphere. Periodic weighting was performed to determine the rate of water vapor movement through the specimen into the desiccant.

In vitro mucoadhesion time of samples was evaluated by assessing the time for samples to detach from the porcine buccal mucosa. The tests were based on modified method of the previous work [40]. The porcine buccal mucosa was bought from a local butcher house and fixed on the internal side of a well-stirred beaker containing 50 mL of artificial saliva maintained at 37 °C. The samples were wetted with artificial saliva and adhered to buccal mucosa. The time for detachment of the samples from buccal mucosa was recorded and taken as in vitro mucoadhesion time.

The tensile strength, Young’s modulus and elongation at break of dry and hydrated films were measured by an INSTRON 5567 Universal Testing Machine (INSTRON, Norwood, MA, USA) equipped with a 1.0 kN load cell. Hydrated samples were prepared by immersing dry films in DI water for 24 h prior to testing. The samples were cut into strip-shaped specimens with a width of 10 mm and a length of 10 cm. The test conditions in this assay followed ASTM D882. The tensile strength, Young’s modulus and elongation at break values were reported as average values determined from at least six specimens.

Human keratinocyte (HaCaT), human oral cancer (CAL-27) cell lines and primary human gingival fibroblasts (GF) were used to evaluate in vitro cytocompatibility as a direct contact test. Human primary gingival fibroblasts (GF, passage 2) were kindly provided by the Department of Anatomy, Faculty of Dentistry, Chulalongkorn University, Thailand. All cell types were cultured in DMEM supplemented with 10% *v*/*v* FBS and 1% of antibiotics in a humidified 37 °C incubator containing 5% (*v*/*v*) CO_2_. The cell cultures of 5 × 10^4^ cells/well were seeded on each film placed in 24-well plates. Cells were allowed to proliferate for 48 h. Cells cultured on glass coverslips were served as control groups. The relative cell viability was evaluated by MTT assay. The optical densities were measured by a Multiskan EX microplate reader (Thermo Scientific, West Columbia, SC, USA) at wavelength of 570 nm. To observe cell morphology on samples, films containing cells were washed with PBS and fixed in 2.5 vol % fixative solution. The samples were dehydrated in serial dilutions of ethanol afterwards and dried in an automated Leica EM-CPD300 critical point dryer (Leica Microsystems, Wien, Austria). Samples were then sputter-coated with gold and observed under a JSM-5410 Scanning Electron Microscope (JEOL, Tokyo, Japan).

Antibacterial testing was carried out according to Japanese International Standard, JISZ2801 [56]. The antibacterial activity analysis of all composite films was tested against Gram-positive *S. aureus* (ATCC 29213) and Gram-negative *E. coli* (ATCC 25922). Briefly, bacteria were suspended in nutrient broth medium. After the cultivation, the bacterial suspensions were diluted with some amount of sterilized water to obtain a suspension with the range of bacteria concentration of 1.6−3.1 × 10^6^ cfu/mL. The bacteria suspension of 0.040 mL was dropped on the surface of each sample, which was horizontally placed on a sterilized petri dish. Each sample size was 5 × 5 cm^2^. The samples were subsequently placed in an incubator and were incubated for 48 h at 37 °C and relative humidity of 90%. After 48 h of incubation, the surfaces of samples were rinsed to harvest bacteria exposed to the samples. The numbers of surviving bacteria on the surface of samples were counted after 48 of incubation by the standard agar plate colony counting method. The images of the agar plates were captured. The above experiments were done in triplicate.

All results were reported as mean ± standard deviation. Statistical analysis was performed using Minitab 17 software (Minitab Inc., State College, PA, USA). The level of statistical significance for all tests was set at *p*-value ≤ 0.05.

## 5. Conclusions

In this study, to develop a multifunctional biopolymer composite film, curcumin was integrated into BCAGG composite film. SEM images indicated a uniform structure of the composites. Mechanical properties showed that BCAGG-C films possessed the appreciable strength and flexibility for practical use as wound dressings. Hydrated films could adhere firmly onto the skin. In vitro mucoadhesion time was found in the range of 0.5–6 h with porcine mucosa as model membrane under an artificial saliva medium. The release of curcumin was not observed in the testing media during 48 h immersion in PBS and artificial saliva, where the fluids uptakes were in the range of 100–700%. Despite being trapped within the biopolymer matrix composite, curcumin could possess useful biological activities. The curcumin-loaded films demonstrated antibacterial activities against *E. coli* and *S. aureus* infection. The films showed anticancer activity against oral cancer cells (CAL-27), but non-cytotoxicity to HaCaT and GF cells. Therefore, BCAGG-C films have potential future applications as wound care dressings and oral mucoadhesive patches for periodontitis or oral cancer treatment.
